# Intra- and post-operative acute hemorrhagic complications of Onyx embolization of brain arteriovenous malformations: A single-center experience

**DOI:** 10.3389/fneur.2022.974954

**Published:** 2022-09-23

**Authors:** Xuan Chen, Yiheng Wang, Jinlu Yu

**Affiliations:** Department of Neurosurgery, First Hospital of Jilin University, Changchun, China

**Keywords:** brain arteriovenous malformation, endovascular treatment, hemorrhagic complication, intraoperative, post-operative

## Abstract

**Background:**

The intra- and post-operative acute (within 72 h) hemorrhagic complications of endovascular treatment (EVT) for a brain arteriovenous malformation (BAVM) are disastrous. Thus, further experiential summaries are required to fully understand them.

**Materials and methods:**

This was a retrospective study of 25 patients with consecutive BAVM who were treated *via* EVT with Onyx embolization and suffered intra- and post-operative acute hemorrhage. The clinical and imaging data of the patients were recorded, analyzed, and discussed.

**Result:**

Twenty-five patients were aged 11–70 years (mean, 37.2 ± 16.1 years), of whom 12 were female (48%, 12/25). Of the 25 hemorrhagic complications, 17 (68%, 17/25) were intraoperative, and 8 (32%, 8/25) were post-operative and occurred between 1 and 12 h after EVT. Of 17 intraoperative hemorrhages, 13 (76.5%, 13/17) were due to high-pressure Onyx casting. Of eight post-operative hemorrhages, six (75%, 6/8) were attributed to normal perfusion pressure breakthrough. The degree of nidus Onyx embolization was more than 2/3 or complete in seven (87.5%, 7/8) BAVMs. Draining vein occlusion was observed in eight (32%, 8/25) of 25 BAVMs. After hemorrhage, conservative treatment was administered in 12 (48%, 12/25) cases, and surgical management was performed in other cases. There were eight cases of mortality; the remaining 17 patients had follow-up data. Among them, 15 patients had good outcomes, with Glasgow Outcome Scale scores of 5 and 4, accounting for 60% (15/25).

**Conclusion:**

In EVT for BAVMs, intra- and post-operative acute hemorrhagic complications are disastrous; only 60% of patients have a good outcome. Therefore, high-pressure Onyx casting or casting too much Onyx at one time to pursue a high degree of nidus embolization should be performed cautiously, and primary draining vein occlusion should be avoided. In short, EVT needs to be performed carefully.

## Introduction

Brain arteriovenous malformations (BAVMs) consist of a tangle of arterialized venular vessels without interposing capillaries, presenting with spirally coiled shapes, which form numerous fenestrated channels; they intercommunicate and empty into thin-walled draining veins (1). Endovascular treatment (EVT) can be used for presurgical or preradiosurgical treatment of BAVMs or as a stand-alone procedure for curative purposes (2, 3). The main role of EVT is either to diminish the nidus size or to occlude high-risk features, such as ruptured nidal and perinidal aneurysms (4).

Currently, the use of the Onyx liquid embolic system (Medtronic, Irvine, CA, USA) is popular in EVT for BAVMs (5). However, EVT with Onyx embolization for BAVMs is not always safe and can be associated with many complications, among which intra- or post-operative acute hemorrhage is disastrous (6, 7). Currently, more summaries of experiences from different centers are required to achieve a full understanding of this type of complication. Therefore, this retrospective study investigated this issue in 25 consecutive patients who experienced this type of complication.

## Materials and methods

### Inclusion and exclusion criteria

The inclusion criteria were as follows: EVT of BAVM with Onyx embolization; and hemorrhagic complication during or in the acute phase after EVT, defined as within 72 h after EVT (8, 9). The exclusion criteria were as follows: hemorrhagic complication occurring more than 72 h post-operatively, and BAVM embolization with an embolic agent other than Onyx.

### Preoperative patient data collection

The data included age, sex, clinical presentation, and intracerebral hemorrhage (ICH) score (10).

### BAVM angioarchitecture

**Figure 1:**
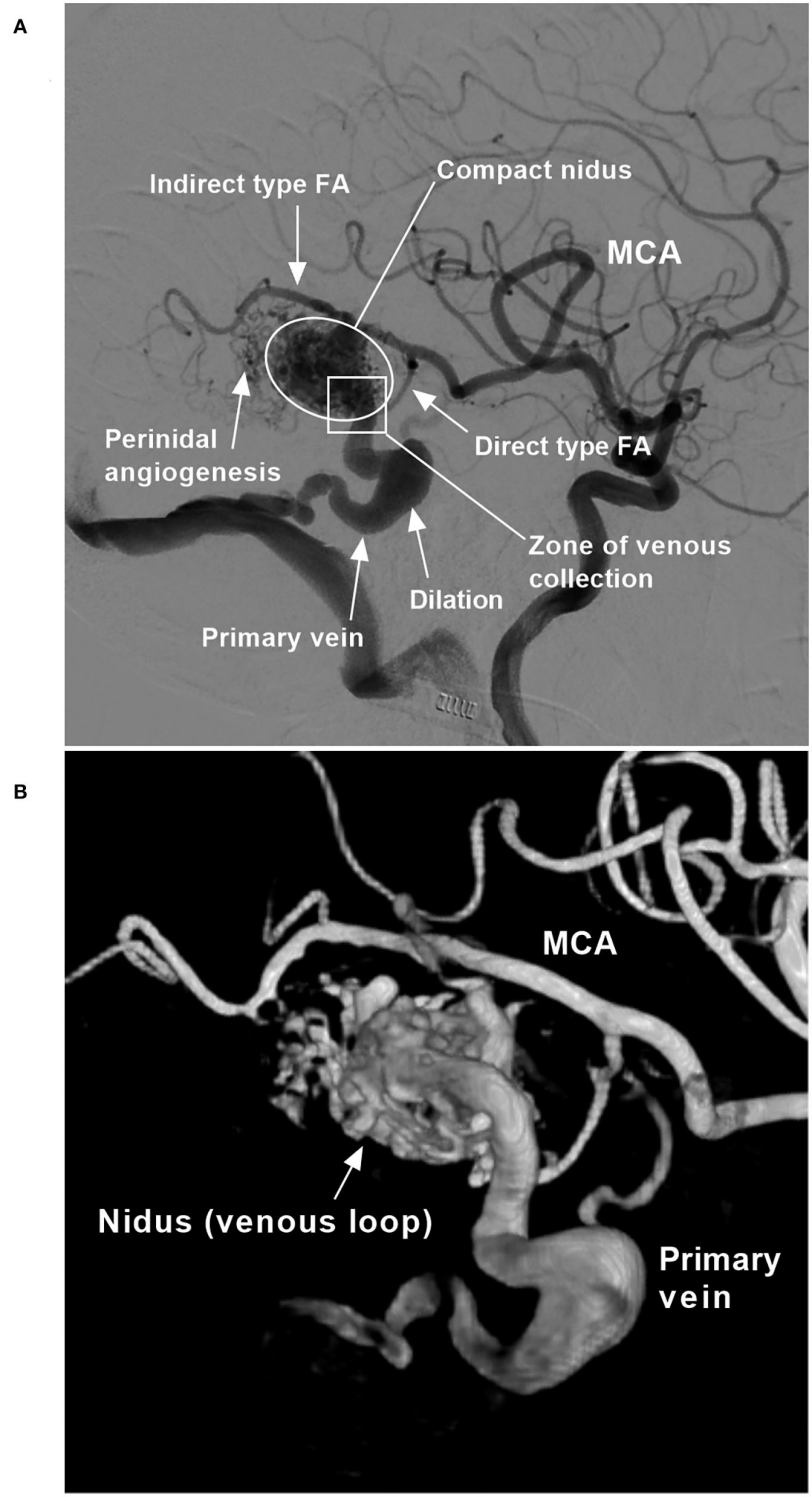
BAVM angioarchitecture (case 6). **(A)** Two-dimensional angiogram. **(B)** Angiogram with a three-dimensional reconstruction. BAVM, brain arteriovenous malformation; FA, feeding artery; MCA, middle cerebral artery.

The location and the Spetzler–Martin (SM) grade of each BAVM were recorded (11). The structural characteristics of the BAVM were recorded, including the feeding artery, the nidus, the draining vein, and any associated aneurysms (Figure 1).

#### Feeding arteries

The origin and remodeling status of the primary and secondary feeding arteries were recorded. The primary feeding artery was the main and largest feeding artery, and the secondary feeding arteries were accessory and thin. Under hemodynamic stress, feeding arteries gradually transition from normal to increasingly abnormal, resulting in remodeling, defined by a diameter two times as large as normal (12).

#### BAVM nidus

A BAVM nidus is a venous component that has a diameter many times larger than the diameter of capillaries, and the size of the nidus was recorded (13). A BAVM nidus can be divided into compact and diffuse types, and the type of the nidus was recorded (1). A compact nidus is defined as a nidus that is wound tightly, with distinct margins. A diffuse nidus is defined as a nidus that is wound loosely, with indistinct margins. The presence of perinidal angiogenesis, which is defined as an angiogenetically induced vascular network within the perinidal brain parenchyma interposed between the feeding artery and the nidus without angiographic evidence of arteriovenous shunts, was also recorded (14, 15).

#### Draining veins

This compartmental vein of the BAVM may exit the nidus either *via* superficial and deep draining veins or *via* primary and secondary draining veins. The primary vein is the largest and collects most of the outflow from the BAVM. The type of draining vein was recorded. Remodeling of the draining vein was also recorded, including the presence of dilation and stenosis (16).

#### Aneurysms and fistulas

Aneurysms associated with each BAVM were recorded; they can be located on either the feeding artery or the draining vein or in the nidus. Aneurysms on the feeding artery present as a saccular dilatation (17). Intranidal aneurysms are usually single and large venous pouches in the nidus (18). Venous aneurysms of the draining venous system present as a spherical or fusiform dilatation (19). An intranidal fistula presents as a feeding artery that is directly connected to the venous drainage system of the BAVM (4).

### Strategy and scheme of EVT

All patients were treated under general anesthesia *via* a transfemoral approach. The Marathon microcatheter, an Apollo microcatheter with a detachable tip, or the Echelon-10 microcatheter (Medtronic, Irvine, California, USA) was used to access the nidus to achieve the wedge position *via* the feeding artery. Then, the Onyx was cast. If further EVT was needed, another feeding artery could be chosen to repeat the Onyx casting. In EVT for BAVMs, weak structures, such as associated aneurysms and fistulous components, should be given priority for treatment. If no weak structure is identified, the main purpose of EVT is to reduce the blood flow of BAVMs to help reduce nidus/perinidal angiogenesis (4).

Flow-related aneurysms on a feeding artery away from the nidus could be treated by coiling embolization; when those aneurysms were close to the nidus, casting Onyx could be applied (20). For intranidal aneurysms, the BAVM compartment containing the aneurysm can be embolized with Onyx casting. For venous aneurysms on the draining vein, the blood flow of the BAVM compartment draining to the vein with aneurysms can be embolized with Onyx casting to reduce the blood flow (21).

**Figure 2:**
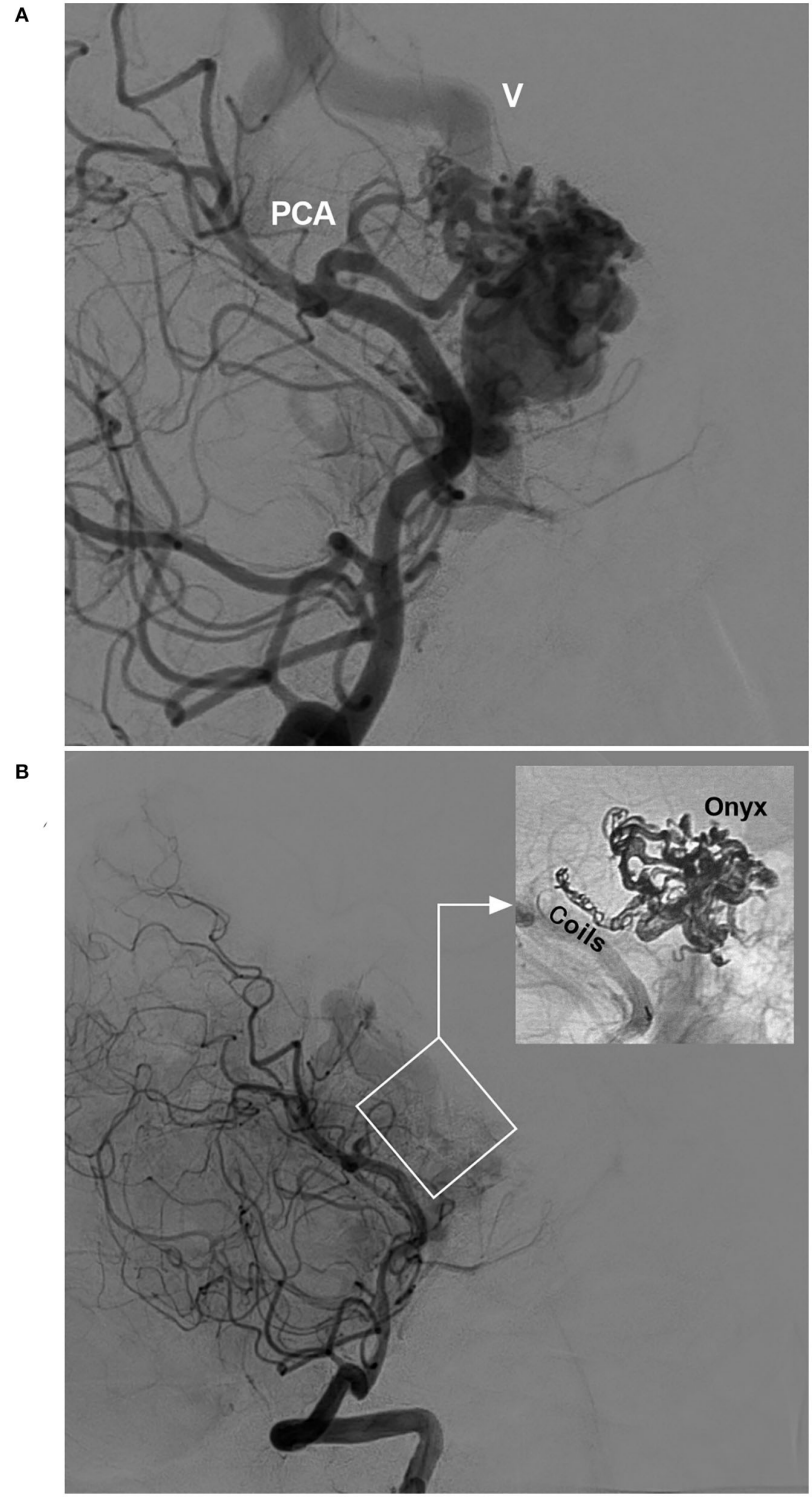
Pressure cooker technique (case 22). **(A)** Angiogram of the VA showing the BAVM supplied by the PCA. Deep vein drainage (V) was indicated. **(B)** Post-operative angiogram of the VA showing nearly complete embolization of the BAVM (frame); demonstration of the pressure cooker technique (inset). Coils were deployed in the feeding artery to prevent Onyx reflux. BAVM, brain arteriovenous malformation; PCA, posterior cerebral artery; VA, vertebral artery.

In EVT for BAVMs, the pressure cooker technique can be used (22). First, a Marathon or Apollo microcatheter is placed in the wedge position in the feeding artery, and then a microcatheter for coiling is placed behind the Marathon or Apollo microcatheter. Before casting Onyx, the feeding artery is coiled to produce the effect of a pressure cooker and avoid reflux during Onyx casting (Figure 2).

### Hemorrhagic complications

**Figure 3:**
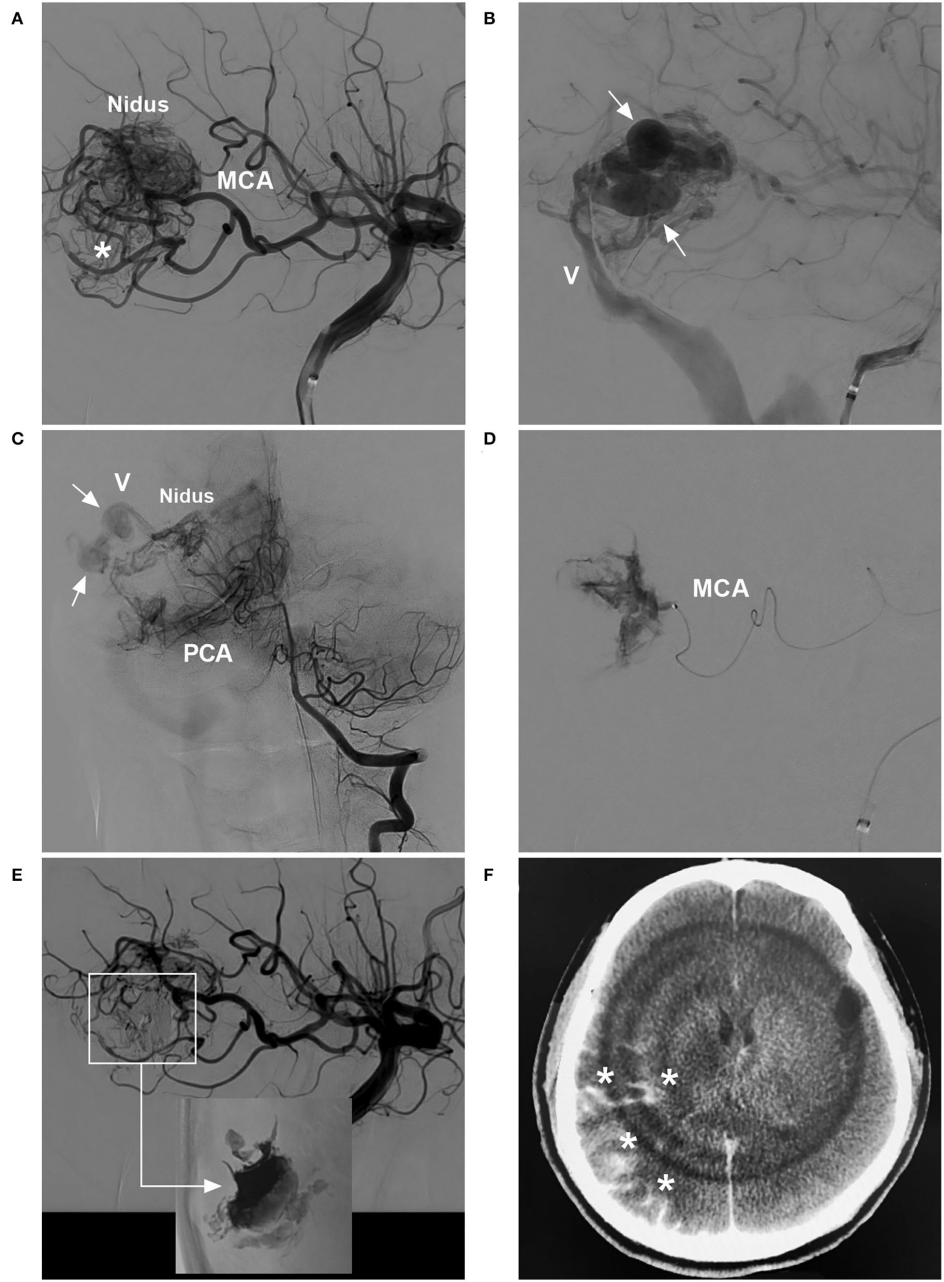
BAVM with intraoperative nidus perforation (case 12). **(A)** Arterial-phase angiogram of the ICA showing the BAVM supplied by the MCA. **(B)** Late arterial-phase angiogram of the ICA showing the draining vein (V) with venous aneurysms (arrows). **(C)** Arterial-phase angiogram of the VA showing the BAVM also supplied by the PCA. **(D)** Selective microcatheter angiogram showing contrast agent extravasation during catheterization and penetration of the nidus. **(E)** Arterial-phase angiogram of the ICA showing the prevention of contrast agent extravasation by continuous embolization (frame); Onyx casting (right-angled arrow) without embolization of the nidus (inset). **(F)** X-per CT showing contrast agent extravasation (asterisks) in subarachnoid space. BAVM, brain arteriovenous malformation; CT, computed tomography; ICA, internal carotid artery; MCA, middle cerebral artery; PCA, posterior cerebral artery; VA, vertebral artery.

**Figure 4:**
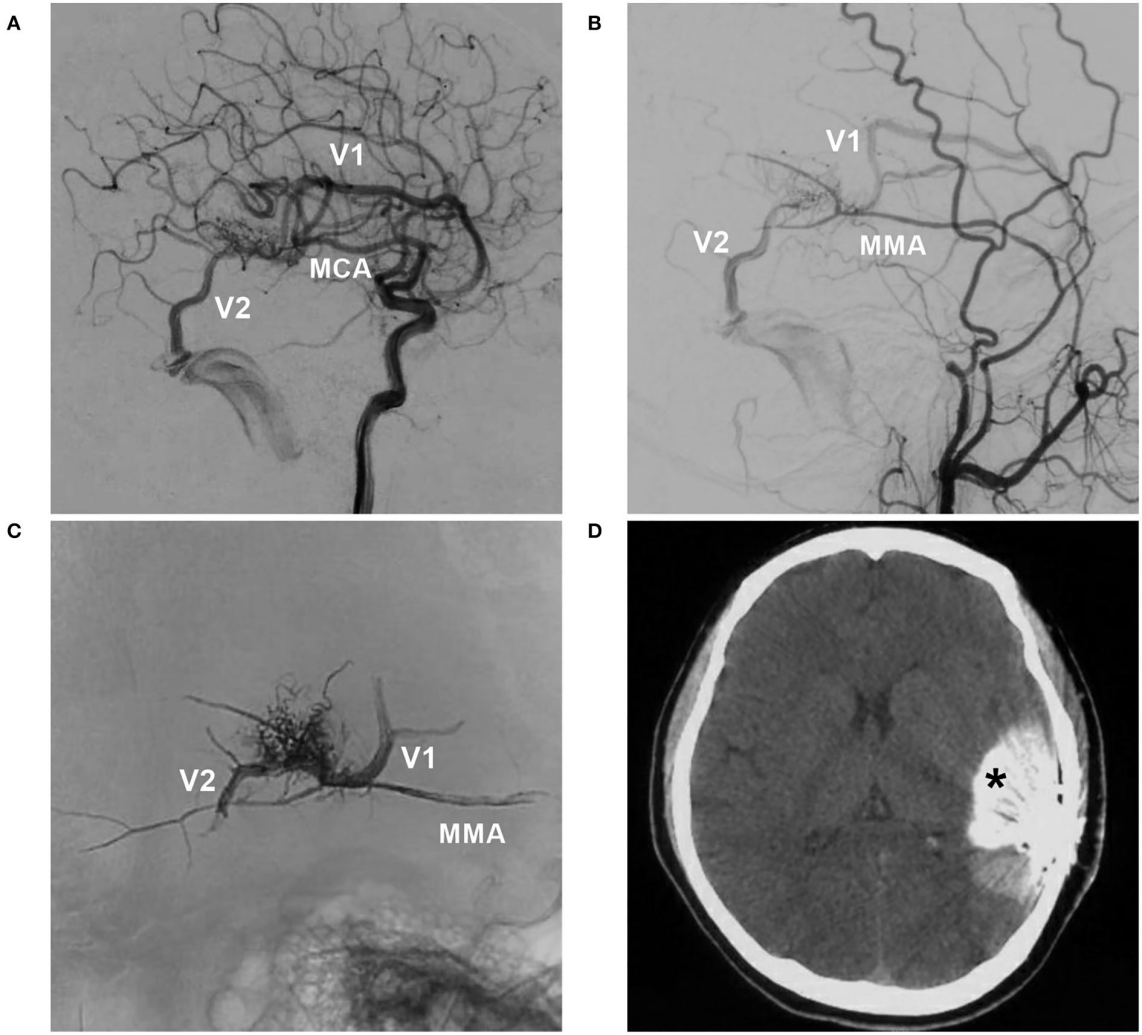
BAVM with intraoperative nidus rupture (case 18). **(A,B)** Arterial-phase angiograms of the internal carotid artery and external carotid artery showing the BAVM supplied by the MCA and MMA, with two draining veins (V1 and V2). **(C)** X-ray film showing Onyx casting *via* the MMA and occlusion of the two draining veins. **(D)** CT showing contrast agent extravasation (asterisk). BAVM, brain arteriovenous malformation; MCA, middle cerebral artery; MMA, middle meningeal artery.

**Figure 5:**
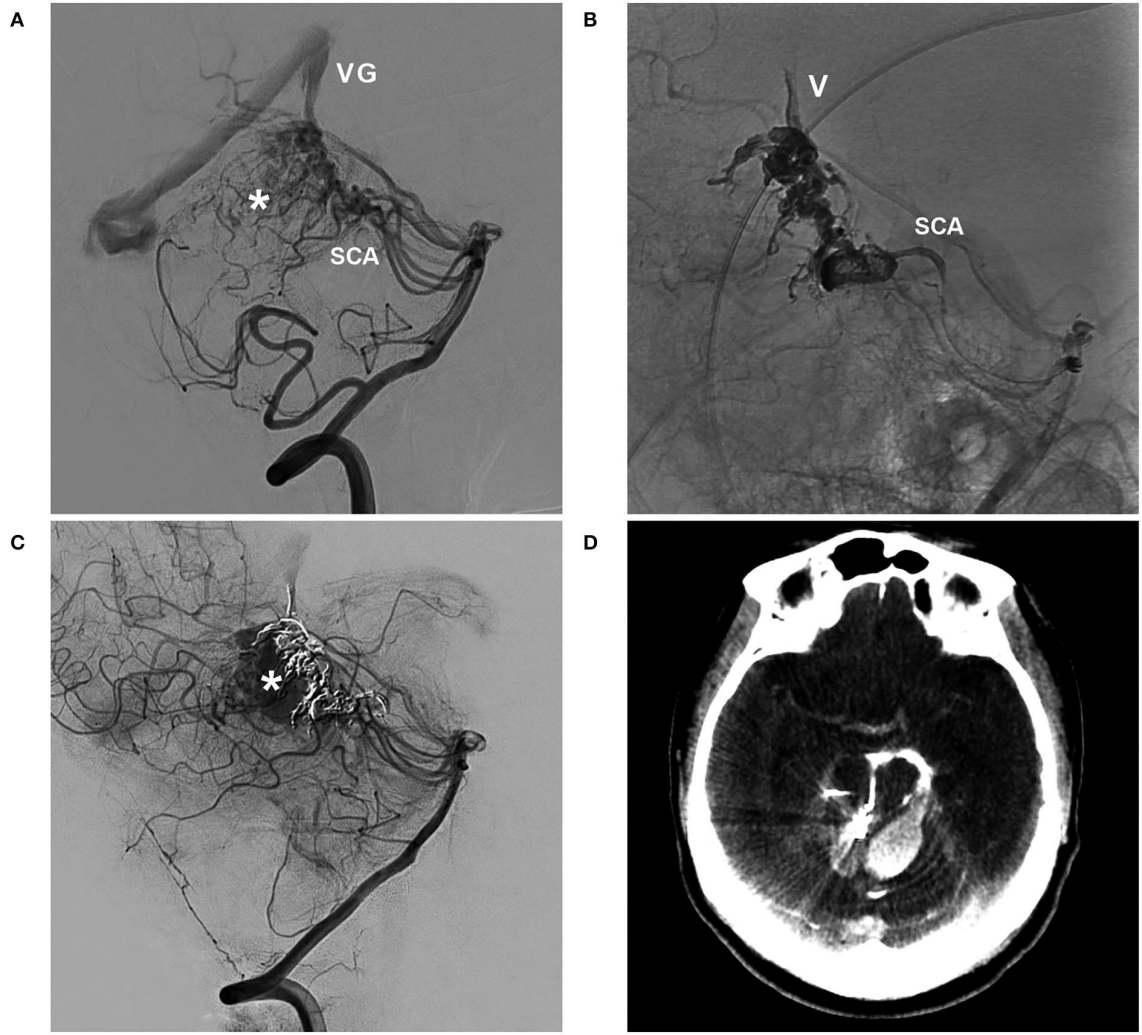
BAVM with intraoperative rupture of area of perinidal angiogenesis (case 1). **(A)** Arterial-phase angiogram of the VA showing the BAVM supplied by the SCA, which drains into the VG; the asterisk indicates perinidal angiogenesis. **(B)** Unsubtracted angiogram of the VA showing Onyx casting in the SCA, nidus, and draining vein (V). **(C)** Angiogram of the VA showing contrast agent extravasation from intraoperative rupture of the area of perinidal angiogenesis (asterisk). **(D)** X-per CT showing contrast agent extravasation. BAVM, brain arteriovenous malformation; CT, computed tomography; SCA, superior cerebellar artery; VA, vertebral artery; VG, vein of Galen.

**Figure 6:**
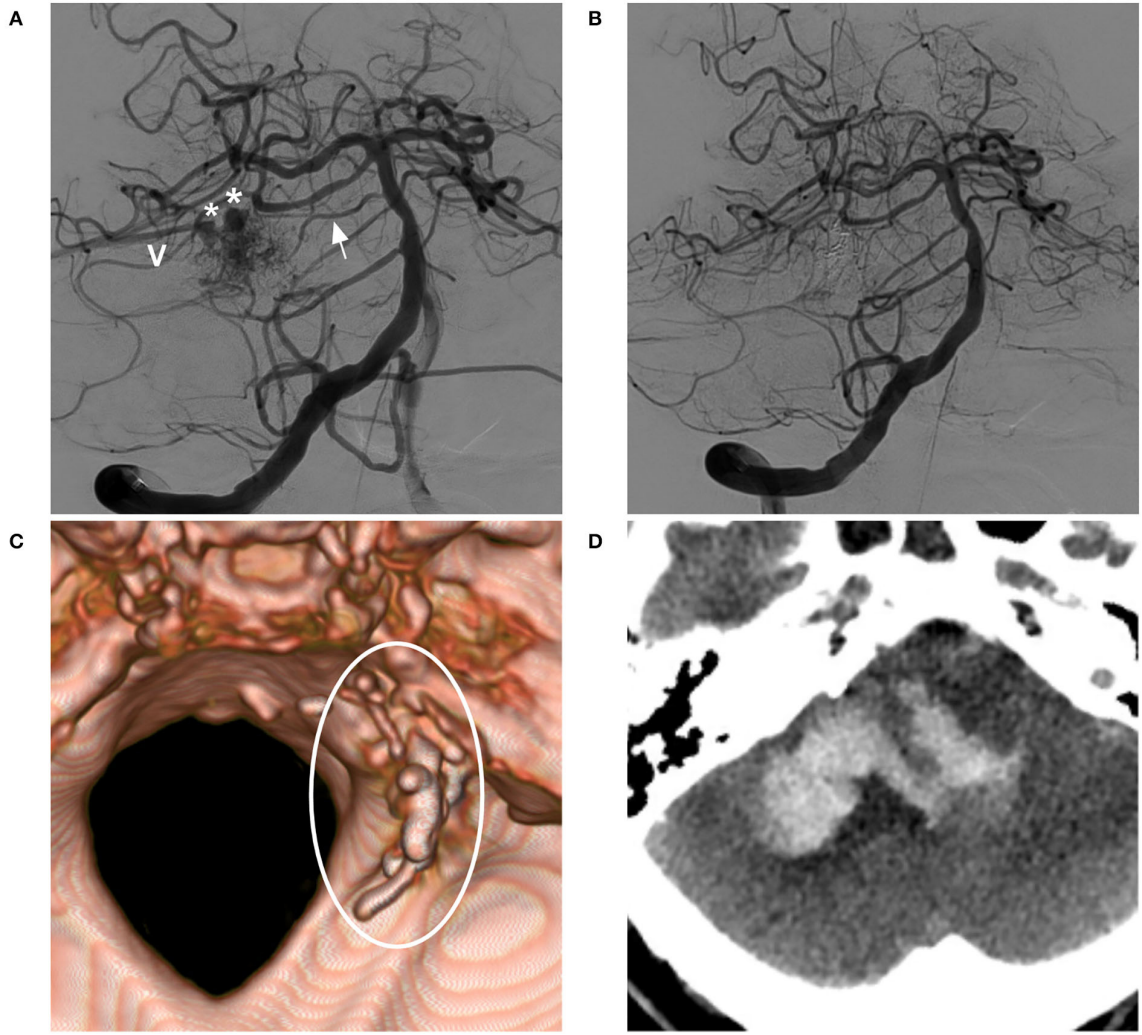
BAVM with post-operative venous rupture (case 13). **(A)** Angiogram of the VA showing the BAVM supplied by the SCA and pontine branch (arrow), with two venous aneurysms (asterisks) on the draining vein (V). **(B)** Post-operative angiogram showing complete embolization of the BAVM and not showing the draining vein. **(C)** Post-operative CT reconstruction showing Onyx casting (circle). **(D)** Post-operative CT showing venous hemorrhage away from the nidus. BAVM, brain arteriovenous malformation; CT, computed tomography; VA, vertebral artery.

**Figure 7:**
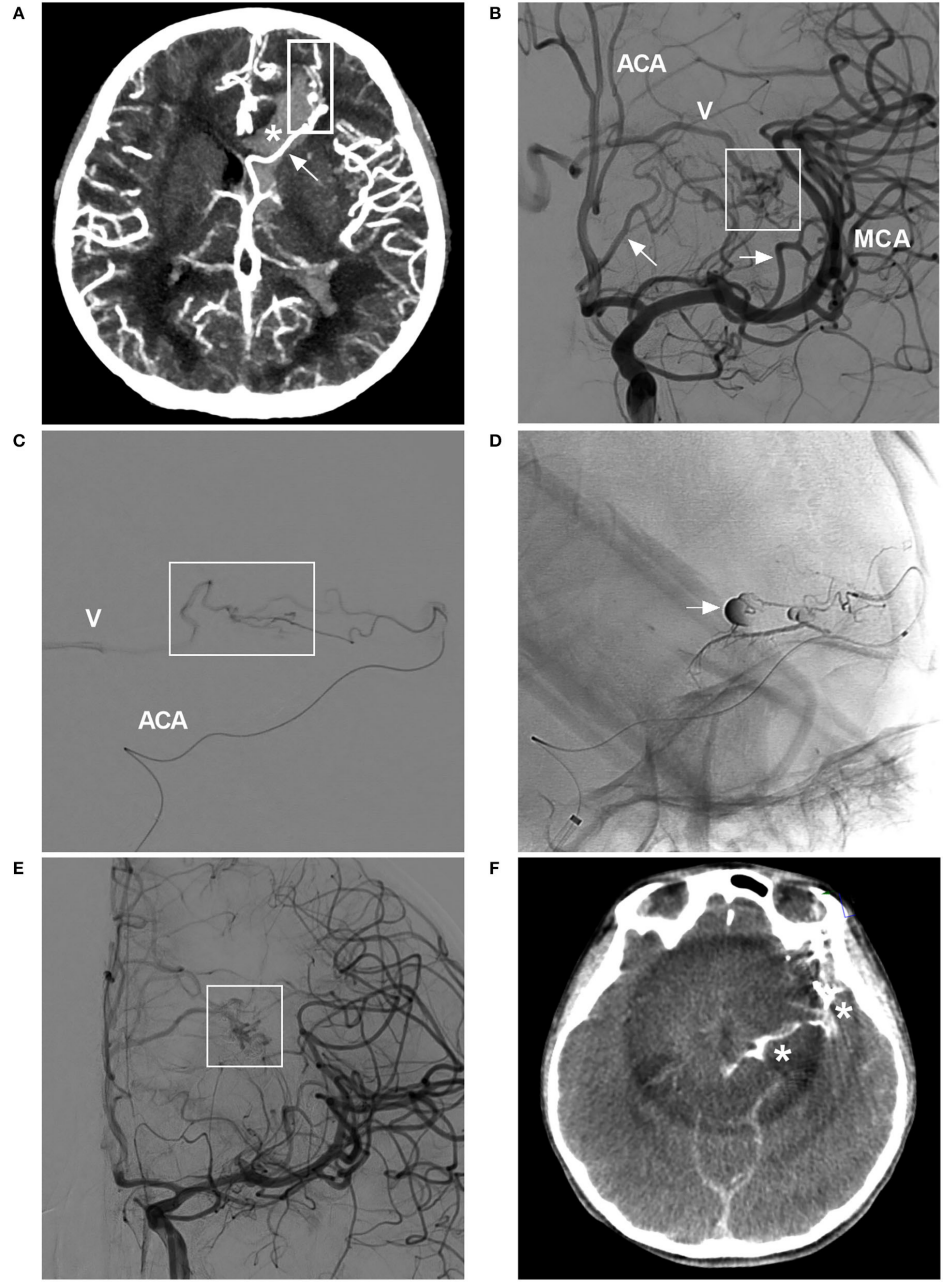
BAVM with intraoperative rupture from Onyx casting (case 25). **(A)** CT angiogram showing BAVM (frame) draining into the deep vein system (arrow), with a rupture into the ventricle (asterisk). **(B)** Angiogram of the ICA showing the BAVM (frame) supplied by the ACA and MCA (arrows), with drainage into the deep vein system (V). **(C)** Selective microcatheter angiogram of the branch of the ACA showing part of the nidus (frame) and draining vein (V). **(D)** Unsubtracted angiogram showing rupture of the nidus during Onyx casting (arrow). **(E)** Angiogram of the ICA showing less residual BAVM nidus (frame) after Onyx casting *via* the ACA and MCA. **(F)** X-per CT showing contrast agent extravasation. ACA, anterior cerebral artery; BAVM, brain arteriovenous malformation; CT, computed tomography; ICA, internal carotid artery; MCA, middle cerebral artery.

**Figure 8:**
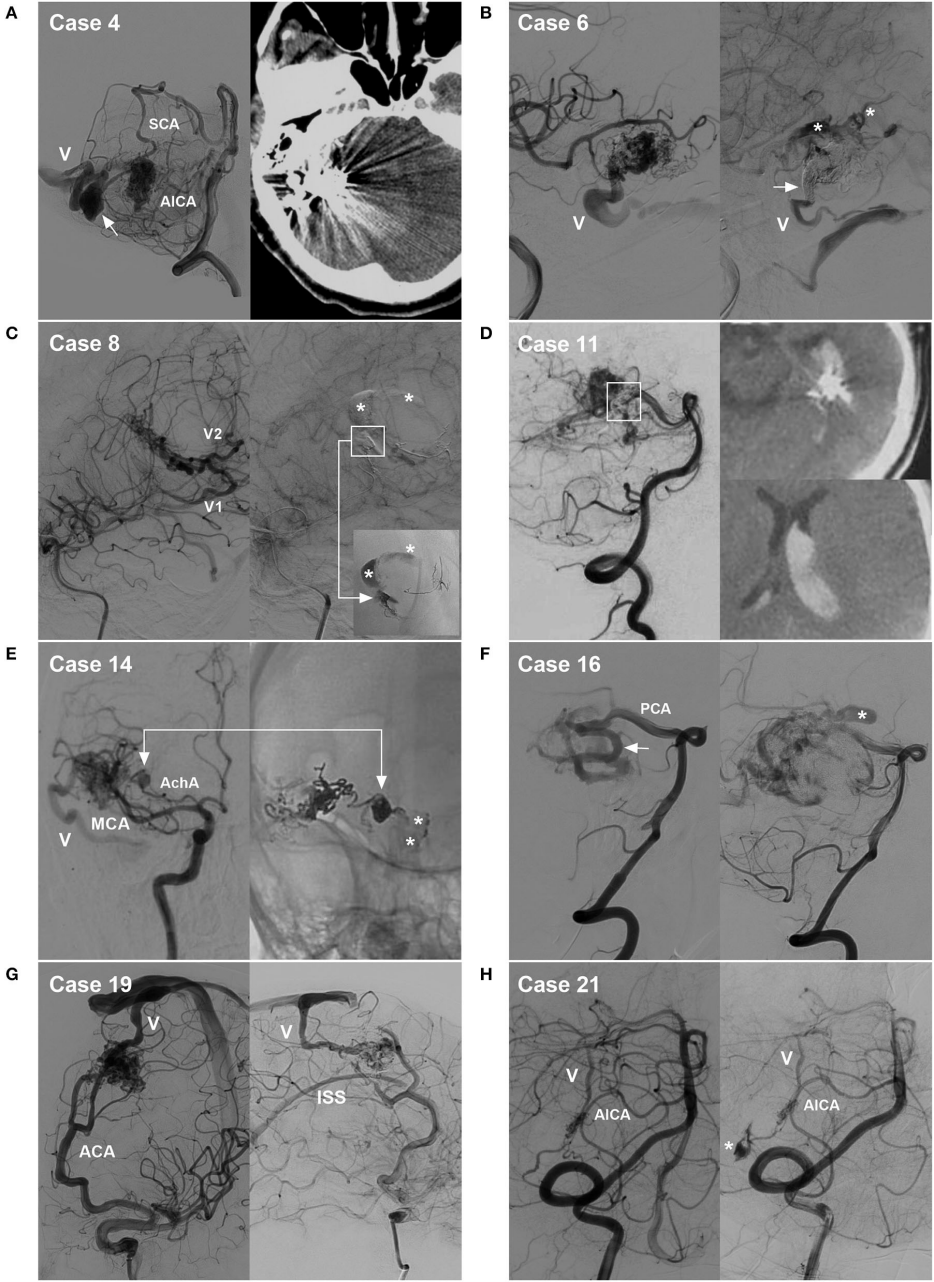
Cases of hemorrhagic complications. **(A)** Draining vein system rupture: left, pre-operative angiogram showing the BAVM supplied by the SCA and AICA, with the draining vein (V) and a large venous aneurysm (arrow); right, post-operative CT showing venous edema and hemorrhage. **(B)** Intraoperative rupture from Onyx casting: left, intraoperative angiogram showing the BAVM supplied by the MCA, with the draining vein (V) to the transverse sinus; right, angiogram of the nidus rupture showing contrast agent extravasation (asterisks) and incomplete occlusion of the draining vein (arrow). **(C)** Intraoperative nidus rupture from Onyx casting: left, pre-operative angiogram showing the BAVM supplied by the MCA, with two draining veins (V1 and V2); right, angiogram of the nidus rupture showing control of the hemorrhage due to continuous embolization. Ruptured point of the nidus (frame); Onyx casting (right-angled arrow, inset); and contrast agent extravasation (asterisks). **(D)** Post-operative hemorrhage from normal perfusion pressure breakthrough: left, post-operative angiogram showing partial nidus embolization (frame); right, post-operative CT showing intraventricular hemorrhage. **(E)** Intraoperative feeding artery aneurysm rupture from Onyx casting: left, pre-operative angiogram showing the BAVM supplied by the MCA and AchA, as well as an aneurysm on the AchA and the draining vein (V); right, angiogram of the aneurysm rupture showing contrast agent extravasation from the aneurysm (arrow, asterisks). **(F)** Non-embolized intranidal aneurysm: left, pre-operative angiogram showing remodeling and dilation of the PCA as the feeding artery (arrow); right, post-operative angiogram showing delayed rupture of the intranidal aneurysm (asterisk) left after embolization. **(G)** Hidden draining vein: left, pre-operative angiogram showing the BAVM supplied by the ACA and draining into the sinus (V); right, post-operative angiogram showing the hidden inferior superior sinus appearing as the draining vein after embolization. **(H)** Arterial perforating complication: left, pre-operative angiogram showing the BAVM supplied by the AICA, with the draining vein (V); right, intraoperative angiogram showing perforation of the feeding artery by the microguidewire (asterisk). ACA, anterior cerebral artery; AchA, anterior choroidal artery; AICA, anterior inferior cerebellar artery; BAVM, brain arteriovenous malformation; CT, computed tomography; MCA, middle cerebral artery; PCA, posterior cerebral artery; SCA, superior cerebellar artery.

For hemorrhagic complications, the imaging characteristics and management strategy were recorded, and the source of the bleeding was determined. Typical and educational cases are provided; these cases and images are from our hospital, and there are no copyright disputes (Figures 2–8).

#### Characteristics and management

Hemorrhagic complications may present as ICH, intraventricular hemorrhage (IVH), or subarachnoid hemorrhage (SAH), among other types, which were recorded. When hemorrhagic complications occurred, the degree of BAVM nidus embolization was recorded as < 1/3, 1/3–2/3, > 2/3, or complete embolization. Whether the draining vein was occluded was also recorded.

When intraoperative hemorrhage occurred, continuous EVT was performed promptly to stop the bleeding. EVT cannot be performed for post-operative hemorrhage. In this case, first, the patients were treated with sedation and controlled hypotension, reducing the systolic pressure to 80–90 mmHg or the average arterial pressure to ~50–65 mmHg (23). For hemorrhage without an occupying effect, conservative treatment was given. For severe hemorrhage with an occupying effect, craniotomy for hematoma evacuation and/or external ventricular drainage (EVD) was recommended accordingly.

#### Determination of the source of bleeding

##### Intraoperative hemorrhage

Bleeding can originate from any part of a BAVM (21). During the navigation of the microguidewire or microcatheter, the feeding artery and nidus can be perforated (Figures 3, 8H). High pressure from Onyx casting can result in intraoperative rupture of the BAVM structure, presenting as Onyx assembly outside the BAVM structure and active contrast agent extravasation (Figure 7). Rupture can be caused by the feeding artery, an aneurysm of the feeding artery (Figure 8E), an intranidal aneurysm, the nidus (Figures 8B,C), or perinidal angiogenesis (Figure 5). When finishing EVT, the feeding artery can rupture from excessive pulling on the microcatheter if the end is stuck (24). All of these conditions are considered technical complications. On post-operative computed tomography (CT), contrast agent extravasation and hemorrhage can be observed in high-density areas. In addition, occlusion of the draining vein during EVT can increase the risk of rupture of the nidus (Figure 4).

##### Post-operative acute hemorrhage

Postoperative hemorrhage can originate from an intranidal aneurysm (Figure 8F), the nidus (Figure 8D), perinidal angiogenesis, or the draining vein system (Figure 8A). On CT after EVT, there may be no contrast agent extravasation. If the BAVM nidus is completely embolized and perinidal angiogenesis is apparent, with hemorrhage around the BAVM, perinidal angiogenesis should be considered the hemorrhagic origin. If the BAVM nidus is not fully embolized, with hemorrhage around the BAVM, the residual nidus should be considered the origin. If the draining vein is occluded, with hemorrhage beside or away from the nidus and associated with edema, venous hemorrhage should be considered (Figure 6).

### Evaluation of the outcomes

The Glasgow Outcome Scale (GOS) score was used to evaluate the outcome at discharge and during the follow-up. The GOS score, ranging from grade 1 to 5, was recorded for all of the patients (25).

## Results

During the period from January 2010 to December 2021, 673 patients diagnosed with a BAVM accepted EVT with 856 Onyx embolization procedures. Of them, 25 experienced intra- or post-operative acute hemorrhage, for a rate of 3.7% (25/673) per patient and 2.9% (25/856) per procedure.

### General and onset information

The patients were aged from 11 to 70 years (mean, 37.2 ± 16.1 years), of whom 12 were female (48%, 12/25). Four (16%, 4/25) patients had an unruptured BAVM; among them, one complained of headache, and three were admitted for epilepsy. Twenty-one (84%, 21/25) patients were admitted for intracranial hemorrhage, including eight patients with ICH, six patients with IVH, five patients with SAH, and two patients with both ICH and IVH. Among the 21 patients with intracranial hemorrhage onset, the ICH score was 1.4 ± 0.7 (score, 0–3).

### Imaging characteristics

#### Feeding arteries

The primary feeding arteries of the 25 BAVMs were as follows: middle cerebral artery, 6 (24%, 6/25); posterior cerebral artery, 6 (24%, 6/25); superior cerebellar artery, 4 (16%, 4/25); posterior choroidal artery, 3 (12%, 3/25); anterior inferior cerebellar artery, 2 (8%, 2/25); anterior cerebral artery, 2 (8%, 2/25); anterior choroidal artery, 1 (4%, 1/25); and middle meningeal artery, 1 (4%, 1/25). Regarding the feeding arteries, remodeling of the primary artery was observed in nine (36%, 9/25) BAVMs (Figure 8F). Of the 25 BAVMs, 14 (56%, 14/25) also had secondary feeding arteries.

#### BAVM angioarchitecture

All 25 BAVM niduses were compact, with a diameter of 2.8 ± 1.1 cm (0.75–5 cm). The locations were as follows: posterior temporal lobe, 5 (20%, 5/25); temporal horn of the lateral ventricle, 4 (16%, 4/25); trigone of the lateral ventricle, 3 (12%, 3/25); cerebellar hemisphere, 3 (12%, 3/25); cerebellar vermis, 2 (8%, 2/25); frontal lobe, 2 (8%, 1/25); body of the lateral ventricle, 1 (4%, 1/25); brainstem, 1 (4%, 1/25); occipital lobe, 1 (4%, 1/25); parietal lobe, 1 (4%, 1/25); basilar ganglia, 1 (4%, 1/25); and posterior thalamus, 1 (4%, 1/25). Perinidal angiogenesis was observed in 11 (44%, 11/25) BAVMs. The SM grades were as follows: grade 1, 2 (8%, 2/25); grade 2, 12 (48%, 12/25); grade 3, 8 (32%, 8/25); and grade 4, 3 (12%, 3/25).

#### Draining veins

The deep vein was used as the primary vein draining model in 12 (48%, 12/25) BAVMs, and the superficial vein was used in 13 (52%, 13/25). Remodeling of the primary draining vein was observed in 14 (56%, 14/25) BAVMs, of which 12 draining veins showed dilatation and two draining veins showed both dilatation and stenosis. Of the 25 BAVMs, 10 (40%, 10/25) fit the secondary draining vein model, while a hidden draining vein was observed in one patient, appearing after EVT (Figure 8G).

#### Associated aneurysms and fistulas

Of the 25 BAVMs, 13 (52%, 13/25) were associated with aneurysms; three had aneurysms on the feeding artery, six had intranidal aneurysms, and four had aneurysms on the draining vein. No fistulas associated with BAVMs were found.

### Results of hemorrhagic complications

Of the 25 patients, two patients accepted EVT with Onyx embolization before admission. Of the 21 patients with ruptured BAVMs, the period between onset and EVT was < 1 day in five patients, 2 days in six patients, 3 days in seven patients, 4 days in one patient, and 5 days in two patients. Of the 25 BAVMs, 17 (68%, 17/25) required a single EVT procedure, and eight (32%, 8/25) required two EVT procedures; overall, 33 procedures were performed. Of them, the Marathon was used in 28 procedures, an Apollo detachable microcatheter was used in four procedures, and the Echelon-10 was used in one procedure. The pressure cooker technique was applied in four (12.1%, 4/33) procedures.

Seventeen (68%, 17/25) of the hemorrhagic complications were intraoperative, and eight (32%, 8/25) were post-operative, occurring between 1 and 12 h (mean, 4.8 ± 3.6 h) after EVT. The hemorrhagic complications included ICH in seven (28%, 7/25) patients, IVH in six (24%, 6/25), both ICH and IVH in five (20%, 5/25), SAH in two (8%, 2/25), both SAH and ICH in four (16%, 4/25), and SAH, ICH, and IVH in one (4%, 1/25).

The hemorrhagic origin in the 17 intraoperative cases included four (23.5%, 4/17) perforating complications (two feeding artery perforations, one feeding artery aneurysm perforation, and one nidus perforation) and 13 (76.5%, 13/17) ruptures of the BAVM structure from high-pressure Onyx casting (10 nidus ruptures, one intranidal aneurysm rupture, one feeding artery aneurysm rupture, and one perinidal angiogenesis rupture). The hemorrhagic origin in the eight post-operative cases included three (37.5%, 3/8) nidus ruptures, two (25%, 2/8) intranidal aneurysm ruptures, one (12.5%, 1/8) perinidal angiogenesis rupture, and two (25%, 2/8) draining vein system ruptures.

When a BAVM ruptured, of the 17 intraoperative hemorrhages, the degree of nidus embolization with Onyx was < 1/3 of the nidus in four (23.5%, 4/17) BAVMs, 1/3–2/3 of the nidus in four (23.5%, 4/17), more than 2/3 of the nidus or the complete nidus in five (29.4, 5/17), and no embolization of the nidus in four (23.5%, 4/17) due to perforating complications. Of the eight post-operative hemorrhages, the degree of the nidus embolization with Onyx was < 1/3 of the nidus in one (12.5%, 1/8) BAVM and more than 2/3 of the nidus or the complete nidus in seven (87.5%, 7/8).

Among the 13 associated aneurysms, Onyx embolization was performed for three feeding artery aneurysms and four intranidal aneurysms, Onyx embolization failed in two intranidal aneurysms, and Onyx casting was performed to reduce the blood flow to four venous aneurysms on draining veins.

Of the 25 BAVMs, eight (32%, 8/25) showed varying degrees of primary draining vein occlusion, including complete occlusion of the primary vein in one BAVM, complete occlusion of both the primary and secondary veins in two BAVMs, incomplete occlusion of the primary vein in four BAVMs, and incomplete occlusion of the primary vein and complete occlusion of the secondary veins in one BAVM. Of the eight occluded primary draining veins, four were associated with intraoperative hemorrhage, and four were associated with post-operative hemorrhage.

Of 17 BAVMs associated with intraoperative hemorrhage, continuous Onyx casting to stop the bleeding was applied in 16 BAVMs, and no management was given in one BAVM (Figure 5). After hemorrhage, conservative treatment was administered to 12 (48%, 12/25) patients, EVD was applied in seven (28%, 7/25) patients, craniotomy was performed in three (12%, 3/25) patients, and craniotomy and EVD were performed in three (12%, 3/25) patients.

### Outcome and follow-up

At discharge, seven (28%, 7/25) patients had a GOS score of 5, five (20%, 5/25) patients had a score of 4, four (16%, 4/25) patients had a score of 3, one (4%, 1/25) patient had a score of 2, and eight (32%, 8/25) patients were deceased, with a score of 1. Among the 17 surviving patients, the follow-up period ranged from 1 to 132 months (mean, 43.8 ± 36.3 months). Eleven of these patients had a GOS score of 5, four patients had a score of 4, one patient had a score of 3, and one patient had a score of 2, with a good outcome (GOS score of 5 or 4) in only 60% (15/25). The detailed clinical and imaging data are shown in Tables 1, 2.

**Table 1:** Clinical data of this study.

**No**.	**Age/sex**	**Onset**	**ICH score**	**Pre-EVT (days)**	**EVT path**	**Hemorrhagic complication**						**Subsequent treatment**	**Post-Operative GOS score**	**Follow-Up and GOS score**
						**Time**	**Type**	**Origin**	**Degree of nidus embolization**	**Draining vein occlusion**	**Intraoperative management**			
1	49/M	ICH	1	2	Single EVT *via* SCA	Intraoperative	SAH and ICH	Perinidal angiogenesis	>2/3 embolization	Incomplete occlusion of the primary vein	No	Conservative treatment	1	–
2	29/M	IVH	1	3	Two EVTs *via* MMA and PCA; PCT	2 h after EVT	ICH and IVH	Intranidal aneurysm	>2/3 embolization without intranidal aneurysm occlusion	Incomplete occlusion of the primary vein; occlusion of the secondary veins	–	Craniotomy and EVD	1	–
3	32/F	SAH	2	< 1	Single EVT *via* AICA	12 h after EVT	ICH and IVH	Perinidal angiogenesis	Complete embolization; aneurysm coiling	Complete occlusion of the primary vein	–	EVD	1	–
4	53/M	ICH	2	3	Single EVT *via* SCA	3 h after EVT	ICH and IVH	Draining vein	>2/3 embolization	Incomplete occlusion of the primary vein	–	Craniotomy and EVD	3	55 months; 4
5	23/M	Epilepsy	–	–	Two EVTs *via* MCA and PchA	Intraoperative	SAH, ICH, and IVH	Nidus	1/3–2/3 embolization	No	Continuous EVT	Conservative treatment	5	76 months; 5
6	67/F	SAH	1	5	Single EVT *via* MCA	Intraoperative	SAH and ICH	Nidus	1/3–2/3 embolization	Incomplete occlusion of the primary vein	Continuous EVT	Conservative treatment	3	77 months; 4
7	12/F	IVH	2	2	Single EVT *via* PchA	Intraoperative	ICH	Nidus	>2/3 embolization	No	Continuous EVT	Craniotomy	4	132 months; 5
8	16/M	ICH	2	< 1	Two EVTs *via* MCA	Intraoperative	ICH	Nidus	< 1/3 embolization	No	Continuous EVT	Craniotomy	4	64 months; 5
9	55/F	IVH	3	5	Single EVT *via* PchA	Intraoperative	IVH	Nidus	< 1/3 embolization	No	Continuous EVT	EVD	1	–
10	35/M	IVH (previous EVT)	1	3	Two EVTs *via* ACA and AchA	Intraoperative	IVH	Nidus	1/3–2/3 embolization	No	Continuous EVT	Conservative treatment	5	74 months; 5
11	32/M	Epilepsy	–	–	Single EVT *via* PCA	5 h after EVT	IVH	Nidus	< 1/3 embolization	No	–	Conservative treatment	5	61 months; 5
12	18/M	ICH	1	< 1	Single EVT *via* MCA	Intraoperative	ICH	Nidus perforation	0	–	EVT to stop bleeding	Craniotomy	5	12 months; 5
13	56/M	SAH	1	2	Two EVTs *via* SCA and pontine branch	8 h after EVT	ICH and IVH	Draining vein	Complete embolization	No	–	EVD	2	5 months; 2
14	36/F	SAH	1	< 1	Two EVTs *via* AchA and MCA	Intraoperative	IVH	Feeding artery aneurysm	< 1/3 embolization	No	Continuous EVT	EVD	4	35 months; 5
15	47/M	Headache	–	–	Single EVT *via* MCA; PCT	Intraoperative	ICH	Nidus	Complete embolization	Complete occlusion of the primary and secondary veins	Continuous EVT	Conservative treatment	5	16 months; 5
16	36/F	Epilepsy	–	–	Single EVT *via* PCA	1 h after EVT	ICH and IVH	Intranidal aneurysm	>2/3 embolization without intranidal aneurysm occlusion	No	–	Craniotomy and EVD	3	16 months; 3
						**Time**	**Type**	**Origin**	**Degree of nidus embolization**	**Draining vein occlusion**	**Intraoperative management**			
17	38/M	IVH	1	4	Two EVTs *via* PCA	Intraoperative	IVH	Intranidal aneurysm	< 1/3 embolization	No	Continuous EVT	EVD	3	3 months; 4
18	38/F	ICH (previous EVT)	0	3	Single EVT *via* MMA	Intraoperative	ICH	Nidus	Complete embolization	Complete occlusion of the primary and secondary veins	Continuous EVT	Conservative treatment	4	58 months; 5
19	70/F	ICH	1	2	Single EVT *via* ACA; PCT	3 h after EVT	ICH	Nidus	>2/3 embolization	No	–	Conservative treatment	1	–
20	28/F	ICH	1	3	Single EVT *via* PCA	Intraoperative	ICH	Nidus	Complete embolization	No	Continuous EVT	Conservative treatment	5	54 months; 5
21	34/F	ICH	2	< 1	Single EVT *via* AICA	Intraoperative	SAH and ICH	Feeding artery perforating	0	–	EVT to stop bleeding	EVD	1	–
22	47/F	IVH	1	3	Single EVT *via* PCA; PCT	4 h after EVT	IVH	Nidus	>2/3 embolization	Incomplete occlusion of the primary vein	–	EVD	1	–
23	49/M	SAH	1	3	Single EVT *via* SCA	Intraoperative	SAH	Feeding artery aneurysm perforation	0	–	EVT to stop bleeding	Conservative treatment	1	–
24	20/F	ICH+IVH	3	2	Single EVT *via* AchA	Intraoperative	SAH	Feeding artery perforation	0	–	EVT to stop bleeding	Conservative treatment	4	6 months; 4
25	11/M	ICH+IVH	1	2	Two EVTs *via* ACA and MCA	Intraoperative	SAH and ICH	Nidus	1/3–2/3 embolization	No	EVT to stop bleeding	Conservative treatment	5	1 month; 5

ACA, anterior cerebral artery; AchA, anterior choroidal artery; EVD, external ventricular drainage; EVT, endovascular treatment; GOS, Glasgow Outcome Scale; ICH, intracerebral hemorrhage; IVH, intraventricular hemorrhage; MCA, middle cerebral artery; MMA, middle meningeal artery; PCA, posterior cerebral artery; PchA, posterior choroidal artery; PCT, pressure cooker technique; SAH, subarachnoid hemorrhage; SCA, superior cerebellar artery.

**Table 2:** Angiographic data of the BAVMs.

**No**.	**Location**	**SM grade**	**Feeding artery**		**Nidus**		**Venous drainage**		**Associated aneurysm**
			**Origin**	**Remodeling of the primary artery**	**Size**	**Perinidal angiogenesis**	**Involvement**	**Remodeling of the primary vein**	
1	Cerebellar vermis	3	Primary: SCA	No	2 × 3 cm	Yes	Primary: one deep vein	No	No
2	Trigone of the lateral ventricle	3	Primary: PCA; secondary: MMA, MCA and PMA	Yes	3 × 5 cm	No	Primary: one superficial vein; secondary: two superficial veins	Dilation	Intranidal
3	Cerebellar hemisphere	2	Primary: AICA	Yes	2 × 2 cm	Yes	Primary: one superficial vein	No	Feeding artery
4	Cerebellar vermis	2	Primary: SCA; secondary: AICA	Yes	3 × 3 cm	No	Primary: one superficial vein	Dilation	Draining vein
5	Posterior temporal lobe	2	Primary: MCA; secondary: PchA	No	5 × 5 cm	No	Primary: one superficial vein	Dilation	No
6	Posterior temporal lobe	1	Primary: MCA	No	2 × 3 cm	Yes	Primary: one superficial vein	Dilation and stenosis	No
7	Trigone of the lateral ventricle	2	Primary: PchA	No	1.5 × 1.5 cm	No	Primary: one deep vein	No	Intranidal
8	Posterior temporal lobe	2	Primary: MCA	No	2 × 4 cm	Yes	Primary: one superficial vein; secondary: one superficial vein	No	Intranidal
9	Posterior thalamus	3	Primary: PchA	No	2 × 2 cm	Yes	Primary: one deep vein; secondary: one superficial vein	Dilation	No
10	Trigone of the lateral ventricle	4	Primary: PCA; secondary: ACA, PchA and AchA	No	4 × 5 cm	Yes	Primary: one deep vein	Dilation	Draining vein
11	Temporal horn of the lateral ventricle	2	Primary: PCA	No	3 × 3 cm	No	Primary: one superficial vein; secondary: two superficial veins	No	No
12	Occipital lobe	2	Primary: MCA; secondary: PCA	No	4 × 5 cm	Yes	Primary: one superficial vein	Dilation	Draining vein
13	Brain stem	3	Primary: SCA; secondary: pontine branch of basilar artery	No	2 × 3 cm	Yes	Primary: one deep vein	Dilation and stenosis	Draining vein
14	Temporal horn of the lateral ventricle	2	Primary: MCA; secondary: AchA	No	3 × 4 m	Yes	Primary: one superficial vein	Dilation	Feeding artery
15	Parietal lobe	2	Primary: MCA; secondary: PCA	No	3 × 3 m	No	Primary: one superficial vein; secondary: one superficial vein	Dilation	No
16	Posterior temporal lobe	2	Primary: PCA; secondary: OA	Yes	4 × 5 cm	No	Primary: one superficial vein; secondary: one deep vein	Dilation	Intranidal
17	Body of the lateral ventricle	4	Primary: PchA; secondary: ACA	Yes	3 × 3 cm	Yes	Primary: one deep vein	Dilation	Intranidal
18	Posterior temporal lobe	1	Primary: MMA; secondary: MCA	Yes	2 × 3 cm	Yes	Primary: one superficial vein; secondary: one superficial vein	No	No
19	Frontal lobe	3	Primary: ACA	Yes	3 × 3 cm	No	Primary: one superficial vein secondary: one deep vein	Dilation	No
20	Temporal horn of the lateral ventricle	2	Primary: PCA	No	0.5 × 1 cm	No	Primary: one deep vein	No	Intranidal
21	Cerebellar hemisphere	3	Primary: AICA	No	1 × 2 cm	No	Primary: one deep vein	No	No
22	Temporal horn of the lateral ventricle	4	Primary: PCA; secondary: AchA	Yes	3 × 3 cm	No	Primary: one deep vein; secondary: one deep vein	Dilation	No
23	Cerebellar hemisphere	3	Primary: SCA	Yes	0.5 × 1 cm	No	Primary: one deep vein; secondary: one deep vein	No	Feeding artery
24	Basal ganglia	3	Primary: AchA; secondary: LSA	No	2 × 3 cm	No	Primary: one deep vein	No	No
25	Frontal lobe	2	Primary: ACA; secondary: MCA	No	1.5 × 2 cm	No	Primary: one deep vein	No	No

ACA, anterior cerebral artery; AICA, anterior inferior cerebellar artery; BA, basilar artery; LSA, lenticulostriate artery; MCA, middle cerebral artery; MMA, middle meningeal artery; OA, occipital artery; PchA, posterior choroidal artery; PMA, posterior meningeal artery; PCA, posterior cerebral artery; SCA, superior cerebellar artery; SM grade, Spetzler–Martin grade.

## Discussion

Except for SM grade IV or V BAVMs, which are generally monitored by observation unless ruptured, EVT plays an important role in securing high-risk bleeding points (such as flow-related or intranidal aneurysms and fistulous components) or reducing the flow to help shrink the nidus or area of perinidal angiogenesis (4, 26). For SM grade I or II BAVMs, EVT can be used as a curative treatment, resulting in total occlusion of the nidus and filling of the proximal part of the draining vein (27–30). However, curative embolization of BAVMs should be considered an unanticipated benefit of such therapy rather than a goal (31). For SM grade III BAVMs, EVT can reduce the BAVM volume before radiosurgery and address high-risk bleeding points (32–34). When BAVMs have large or deep feeding arteries, pre-operative embolization can be used to reduce the flow greatly, prevent intraoperative bleeding, and reduce local venous hypertension and vascular steal, which facilitates microsurgical resection (35).

However, EVT for BAVMs is a challenge, especially considering hemorrhagic complications, which are the most frequent complication of EVT, reported in 2–12.5% of procedures (6, 36). In a study by Baharvahdat et al., including 846 embolization procedures in 408 patients, hemorrhagic complications occurred in 11% of procedures (6). In a report by Sato et al. with a total of 1042 EVT procedures, the rate of hemorrhagic complications was 5.7% (36). In a report by Liu et al. describing the use of Onyx as an embolic agent in 126 patients with 143 consecutive interventions, the periprocedural bleeding complication rate was 5.4% per patient and 4.7% per procedure (37).

In our study, the rate was 3.4% per patient and 2.9% per procedure, which is lower than that in the above reports. The main reason for this difference is that our study excluded hemorrhagic complications occurring more than 72 h post-operatively, which resulted in the inclusion of fewer cases. At our center, microwire navigation and microcatheter catheterization are performed very cautiously. Therefore, the rate of arterial rupture due to technical complications is not as high as that reported by Baharvahdat et al. in their report, 48% of hemorrhagic complications were related to arterial rupture (6); in our study, the corresponding rate was 16%. In addition, in our study, the pressure cooker technique was applied in 12.1% of procedures. After Onyx casting, due to the lack of a long reflex distance of Onyx, the coiling did not fix the microcatheter, and the microcatheter could be easily retrieved without difficulty, as suggested in our previous reports (38, 39).

High-pressure Onyx casting plays an important role in the intraoperative rupture of BAVMs, and this type of complication should also be considered a technical complication (16). During Onyx casting, due to intercompartmental communications, theoretically, a single Onyx casting may obliterate all compartments of the nidus. However, when continuing the casting to push excess Onyx from one compartment into other compartments, the increased pressure of Onyx casting can lead to rupture of the BAVM structure, especially in cases with intranidal aneurysms or even feeding artery aneurysms (16, 18, 40). In our study, rupture from high-pressure Onyx casting accounted for 76.5% of intraoperative hemorrhages. During Onyx casting, early primary draining vein occlusion will increase the resistance to Onyx casting and increase the risk of intraoperative hemorrhage (41). Among 17 intraoperative hemorrhagic complications, there were four perforating complications; of the remaining 13 complications, there were four primary draining vein occlusions, accounting for 30.8% (4/13). Therefore, primary draining vein occlusion should be avoided.

When a BAVM exhibits a monocompartmental compact nidus with one feeder, elimination of the single feeder leads to the collapse of the entire BAVM (13). However, in our study, with 25 BAVMs, 56% of BAVMs had secondary feeding arteries, and 40% of BAVMs had secondary draining veins, which indicated that many BAVMs were multicompartmental, resulting in increased resistance to Onyx casting and a greater risk of intraoperative hemorrhage (16). Therefore, when there is resistance, EVT should be performed with caution. To reduce the occurrence of intraoperative hemorrhagic complications, Cai et al. proposed simultaneous Onyx casting *via* dual catheters because injection from two different feeders can result in the gradual, centripetal exclusion of the nidus (42). However, more evidence of the technique is required to support its application in clinical practice.

Currently, definite causes of post-operative hemorrhagic complications are unclear. Only some hypotheses have been proposed. According to Baharvahdat et al. post-operative hemorrhagic complications may originate from normal perfusion pressure breakthrough that can result in the rupture of a non-embolized nidus or area of perinidal angiogenesis due to increased hemodynamic stress in this region. Complete or incomplete draining vein occlusion can also result in post-operative rupture of the nidus or area of perinidal angiogenesis due to venous hyperemia or progressive flow slowing or thrombosis of the draining vein. In addition, inflammatory reactions or mural necrosis induced by the embolic material can result in post-operative hemorrhage (6).

In our study, except for two (25%, 2/8) hemorrhages attributed to draining vein system rupture, six (75%, 6/8) post-operative hemorrhages were attributed to normal perfusion pressure breakthrough. The casting of an excessive amount of Onyx at one time can increase the occurrence of post-operative normal perfusion pressure breakthrough (43–47). In a report by Katsaridis et al. 5% (5/101) of patients had post-operative hemorrhagic complications; in these patients, the degree of nidus embolization with Onyx was often more than 80% (48). In our study, among eight post-operative hemorrhages, the degree of nidus embolization with Onyx was more than 2/3 of the nidus or the complete nidus in 87.5% of cases. Therefore, complete embolization of the nidus should not be pursued in EVT for BAVMs (27, 30, 31). Staged embolization with Onyx over 4–6 months can be helpful, with < 30% obliteration of the nidus every time (7, 26).

In our study, of the eight BAVMs with post-operative complications, primary draining vein occlusion occurred in four, accounting for 50%, which increased the risk of post-operative hemorrhagic complications. When the draining function of the primary draining vein is impaired due to unexpected Onyx occlusion or curative embolization, venous congestion in the adjacent brain region might progress through delayed thrombosis in the draining vein, and the scant venous flow may result in delayed occlusion of the remaining drainers, resulting in post-operative venous hemorrhage (3, 49). Our study included two confirmed draining vein system ruptures. Therefore, the procedure of EVT for BAVMs should not compromise venous drainage, especially the plexiform component or dural sinuses of the drainage vein system (50).

Intra- and post-operative acute hemorrhagic complications may be disastrous; in our study, 40% of patients died. For intraoperative hemorrhage, the only treatment available is continuous embolization until the bleeding stops. If the rupture point is small, there is less bleeding, and a good prognosis can be achieved; otherwise, the bleeding can be disastrous or fatal. EVT cannot be performed for post-operative hemorrhage, and the prognosis is determined by the severity and location of the hemorrhage.

## Conclusion

For intraoperative hemorrhagic complications, in addition to vessel rupture-related complications, high-pressure Onyx casting is an important risk factor. For post-operative acute hemorrhagic complications, casting an excessive amount of Onyx at one time to increase the degree of nidus embolization may result in post-operative hemorrhage due to normal perfusion pressure breakthrough. Primary draining vein occlusion can also increase the risk of intra- and post-operative acute hemorrhage. Hemorrhagic complications can be disastrous; in our study, only 60% of patients had a good outcome.

## Limitations

This was a retrospective study without a control group. The conclusion should be interpreted with caution. In addition, the results cannot indicate whether the rupture status of BAVMs prior to EVT is related to the occurrence of post-operative hemorrhagic complications, whether hemorrhage complications tend to occur in infratentorial BAVMs or high SM grade BAVMs, or whether the pressure cooker technique increases hemorrhage complications.

## Data availability statement

The raw data supporting the conclusions of this article will be made available by the authors, without undue reservation.

## Ethics statement

Written informed consent was obtained from the individual(s), and minor(s)' legal guardian/next of kin, for the publication of any potentially identifiable images or data included in this article.

## Author contributions

JY and XC contributed to the conception, design of the manuscript, and critically revised the manuscript. XC and YW wrote the manuscript and collected the medical records of the patients. All authors approved the final version of this manuscript.

## Conflict of interest

The authors declare that the research was conducted in the absence of any commercial or financial relationships that could be construed as a potential conflict of interest.

## Publisher's note

All claims expressed in this article are solely those of the authors and do not necessarily represent those of their affiliated organizations, or those of the publisher, the editors and the reviewers. Any product that may be evaluated in this article, or claim that may be made by its manufacturer, is not guaranteed or endorsed by the publisher.
